# Women's Clothing

**Published:** 1859-01

**Authors:** 


Women's Clothing.

In an article on *Cheap Clothing,* reprinted from the *Englishwoman's Review,* the evils resulting from the use of insufficient clothing are pointed out. Cheap material is, in the opinion of the writer, one of the banes of our female population; inducing consumption and degeneration of race. It is believed, probably with correctness, that England is the only country in Europe, where the dress of the lower class is not appropriate to the climate.

"The peasantry in Normandy and Brittany, though their climate is far better than ours, and the winter much shorter, clothe themselves in substantial woollen garments as cold weather approaches; indeed, they carry a weight of clothing at all seasons which to us appears extraordinary. The costume of the women in these provinces, who come to the markets with country produce, is usually the following:—A close fitting suit with long sleeves, of dark blue cloth, a very full plaited petticoat of dark woollen plaid, a scarlet or yellow shawl of small size, crossed in front, and tightly pinned down, an apron of showy pattern, with large pockets, of some cotton fabric, and passing out from the short upper garment may be seen a red woollen petticoat and thick grey worsted stockings; in addition to this they wear a camisole, or under-vest, close to the throat, with long sleeves of their own knitting; and with the crowning article, the coiffe, which, in Normandy is made up of muslin, lace, and pasteboard, the female peasant looks more picturesque than ours do with their attempts at the fashion of their betters."

